# A novel approach to distal femur: a minimally invasive technique for supracondylar and intercondylar fracture

**DOI:** 10.1186/s13018-022-03076-7

**Published:** 2022-03-24

**Authors:** Tsung-Mu Wu, Chi-Sheng Chien, Sheng-Hui Lin

**Affiliations:** Orthopedic Department, Chi-Mei Medical Center, No. 901, Zhonghua Rd., Yongkang Dist., Tainan City, Taiwan (ROC)

**Keywords:** Distal femur, Supracondylar fracture, Intercondylar fracture, Minimally invasive surgery, Minimally invasive plate osteosynthesis, Novel, Technical note

## Abstract

**Background:**

For treating distal femur fractures, minimally invasive plating techniques with indirect reduction of the metadiaphysis while minimizing the damage to the peripheral soft tissue has gradually become the standard. However, all the current approaches use a straight or lazy curved incision adjacent to the patella or along the lateral side of the femur, which allows for easier proximal extension but increases the incision length.

**Methods:**

In order to achieve a more physiological and esthetic outcome while leaving the metadiaphysis untouched, we developed an approach using a lambda-shaped incision, which sacrifices the potential for proximal extension but preserves much more peripheral soft tissue. Here, we describe our technique and our experience with it in 19 patients (12 men and 7 women).

**Results:**

Fractures healing by first intention was observed in all patients. The postoperative knee range of motion can reach up to 90° in most of the patient. Clinically, 9 patients had excellent results, 6 had good results, 3 had fair results, and 2 had loss of follow-up.

**Conclusions:**

Our minimally invasive lambda-incision approach allows sufficient visual access to almost the entire joint surface, including the entire lateral femoral condyle, trochlear surface, and distal medial condyle, where reduction is required. The lambda incision provides a much larger window than that offered by a same-length straight incision. The vertical turn at the mid-patellar level parallels the skin crease and geniculate artery, reducing soft tissue damage and resulting in a smooth healing wound. Moreover, plate and distal screw insertion is easier than that through a straight incision. Because the metadiaphysis region is mostly left untouched, ideal fracture-healing environment is preserved.

## Introduction

Locking compression plate fixation is the current preferred treatment for patients with distal femur fractures. It has the advantages of a fixed angle structure that maintains the anatomical axis of the knee when treating osteopenic fractures, comminuted fractures, and periprosthetic fractures following total knee arthroplasty. Marcy [1] was the first to describe the lateral approach to treating supracondylar fractures in the distal femur. However, for intercondylar fractures, reduction of the articular surface can be difficult when employing this approach; the incision often needs to be extended much further to subluxate the extensor component medially to expose the articular surface. To address this problem of inadequate joint exposure and modify the lateral approach, Olerud et al. extended the incision distally toward the tibial tubercle and turned it backward to the medial side of knee—forming a J-shaped curve—following a tibial tubercle osteotomy; in this procedure, the entire extensor component, with the skin flap, can be reflected to expose the complete distal femoral joint [2]. These types of extensive approaches, however, have raised concerns regarding the risk of knee devascularization and interference with the future skin incision necessary for total knee arthroplasty (TKA). Starr et al. [3] described the “swashbuckler” approach to the distal femur—it entails a midline incision of the knee and a lateral subvastus arthrotomy; they claimed that this approach not only provides good exposure of the articular surface but also preserves the knee extensor mechanism and a possible route for future TKA [3]. Krettek et al. used a midline incision with lateral parapatellar arthrotomy to treat intraarticular fractures. They emphasized the value of indirect reduction to avoid excessive soft tissue damage around the metadiaphysis [4]. This concept eventually led to the introduction of modern minimally invasive percutaneous plating osteosynthesis techniques. Approaches to the distal femur have been modified to pursue only secure joint reduction with the least amount of soft tissue damage possible while maintaining the gross anatomical alignment.

In 2015, we developed a novel minimally invasive approach to reduce the distal femoral fracture without compromising most of the genicular arteries and knee extensor components. An appropriate retractor placement ensures the exposure of almost the entire distal femoral joint and provides a sufficient lateral surface of lateral femoral condyle for plate and distal screw insertion. Since this technique was developed, Dr. Wu and Dr. Lin have routinely used it to treat patients with closed distal femur periarticular fractures at our institution, and positive results have been obtained. In this article, we described the detail and concept along with some tips and tricks of this technique and demonstrated the objective and clinical outcome of the patients undergone the procedure.

## Methods

### Inclusion and exclusion criteria

We used a diagnostic registry to include only patients who underwent surgical treatment by Dr. Wu and Dr. Lin. The keywords used to filter the patients were “distal femur,” “supracondylar,” and “intercondylar.” Next, we reviewed each case and excluded those with major complications, such as instances of open fractures, brain injury, revision surgery, or dysplastic femur. Pure medial condyle fracture and Hoffa fracture were also excluded because they can be easily treated with other even less invasive approaches.

### Surgical technique

**Fig. 1:**
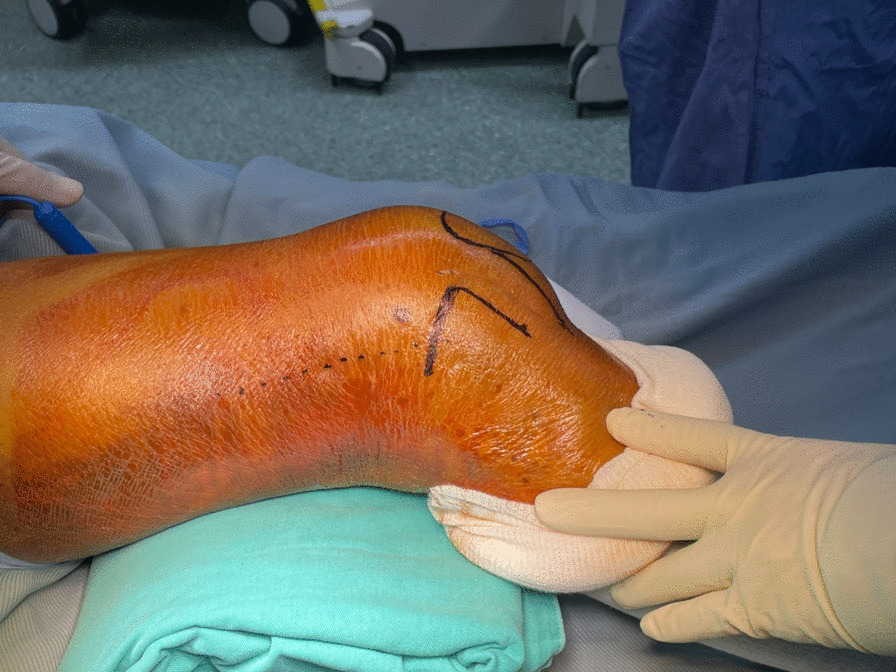
Triangular leg roll positioned beneath the knee

**Fig. 2:**
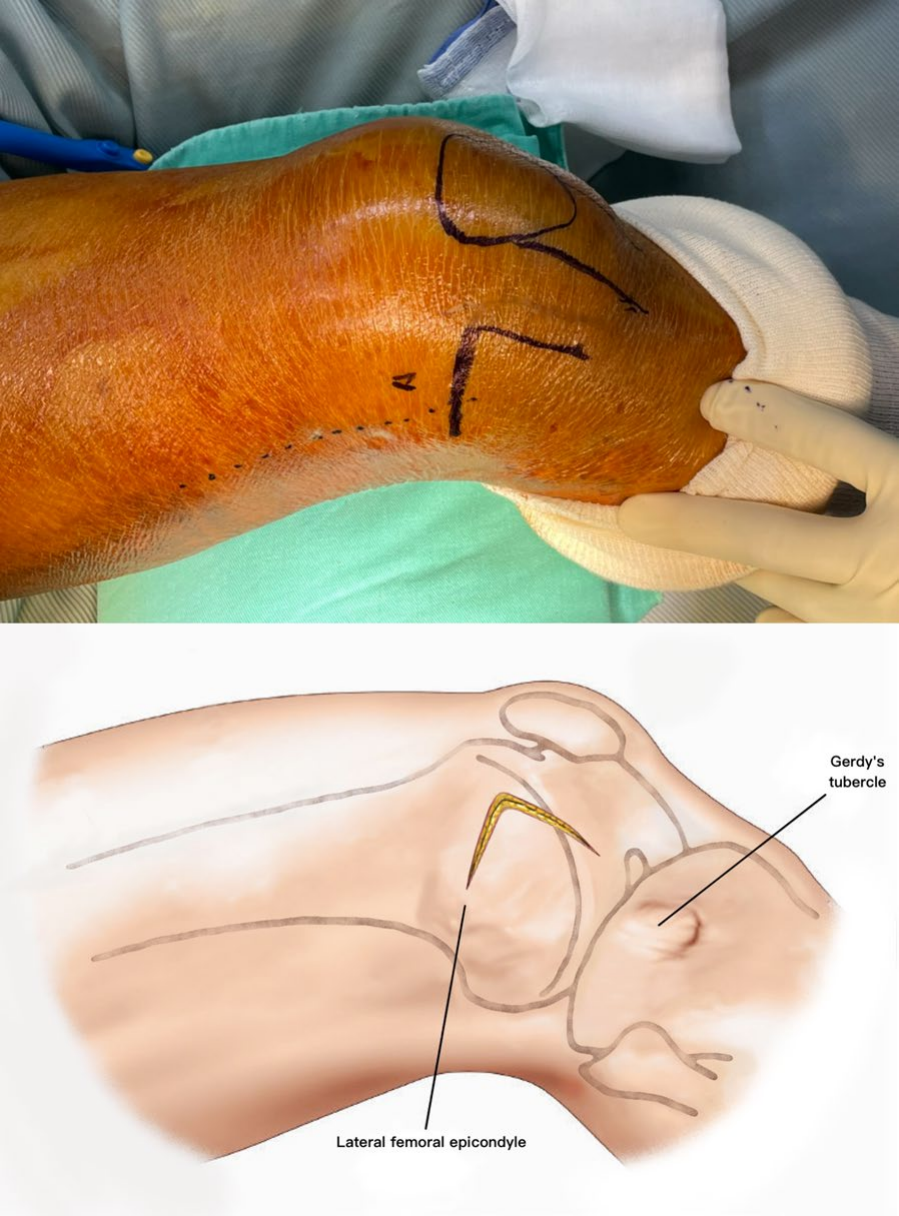
The lambda incision starts from Gerdy’s tubercle and extends proximally parallel to the patellar tendon. A perpendicular turn is made after reaching the mid-patellar level, and the incision is extended toward the lateral femoral epicondyle along the skin crease

**Fig. 3:**
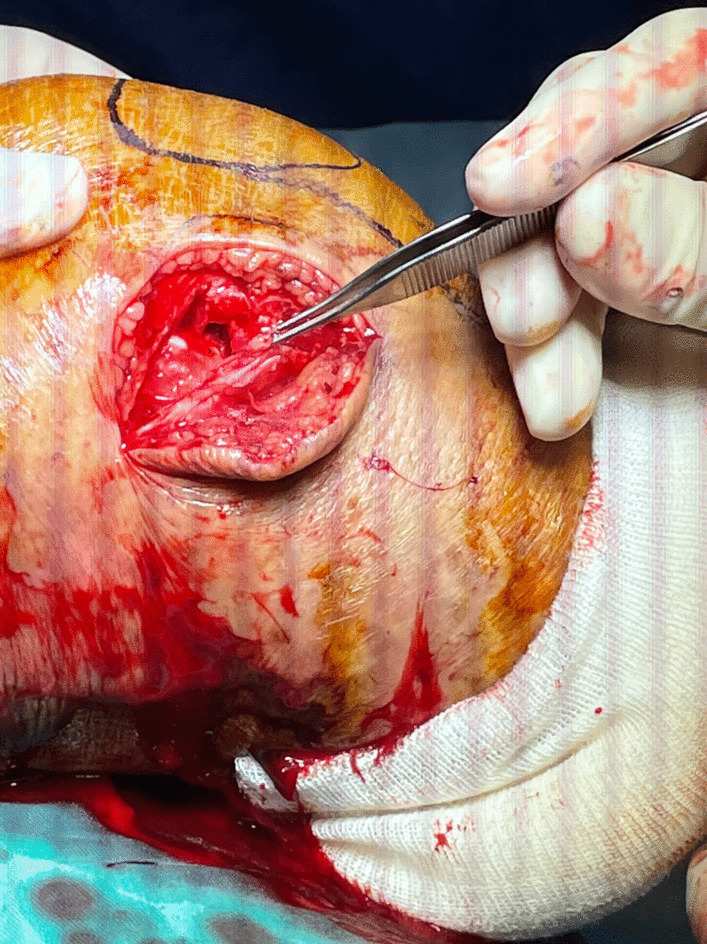
The retinaculum is incised in the same manner as the skin

**Fig. 4:**
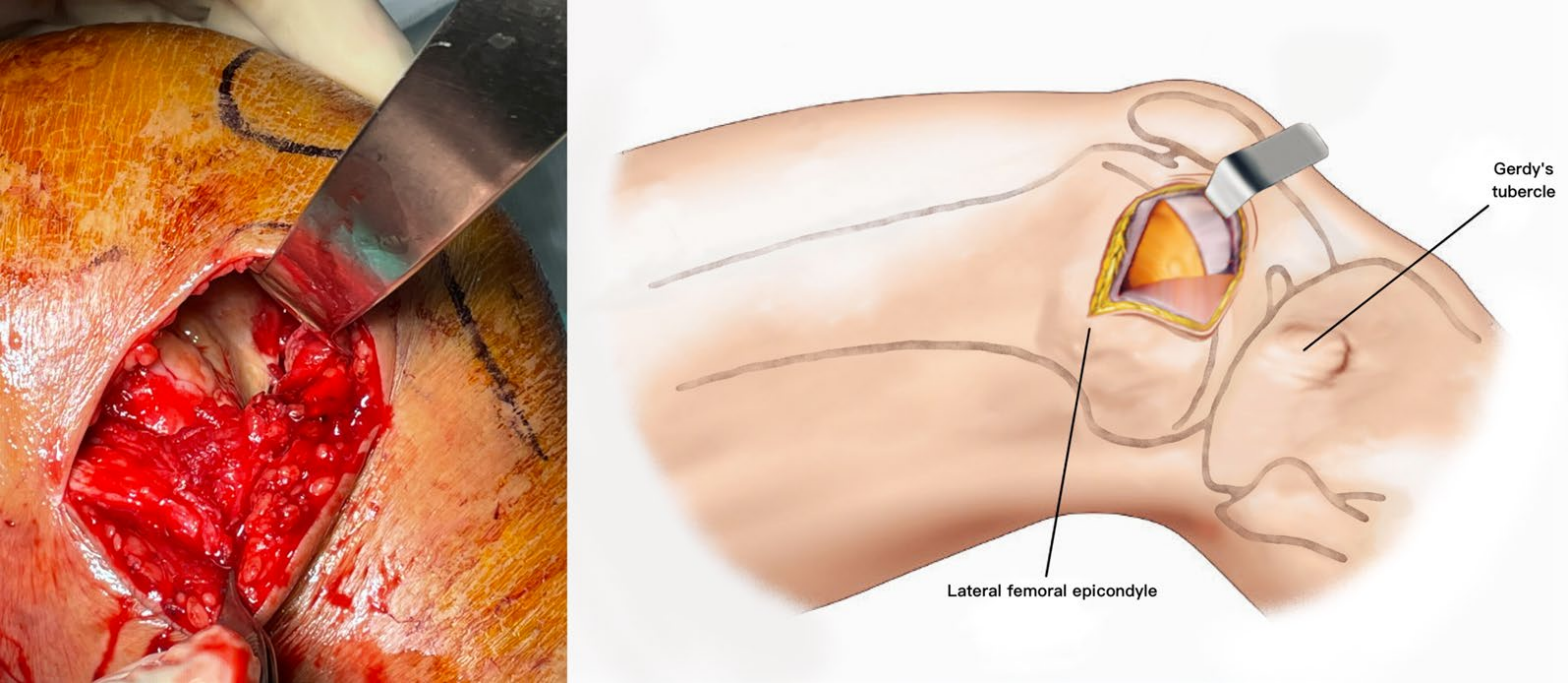
A curved Hohmann retractor is placed across the suprapatellar pouch to render visible the intercondylar notch and joint congruity

**Fig. 5:**
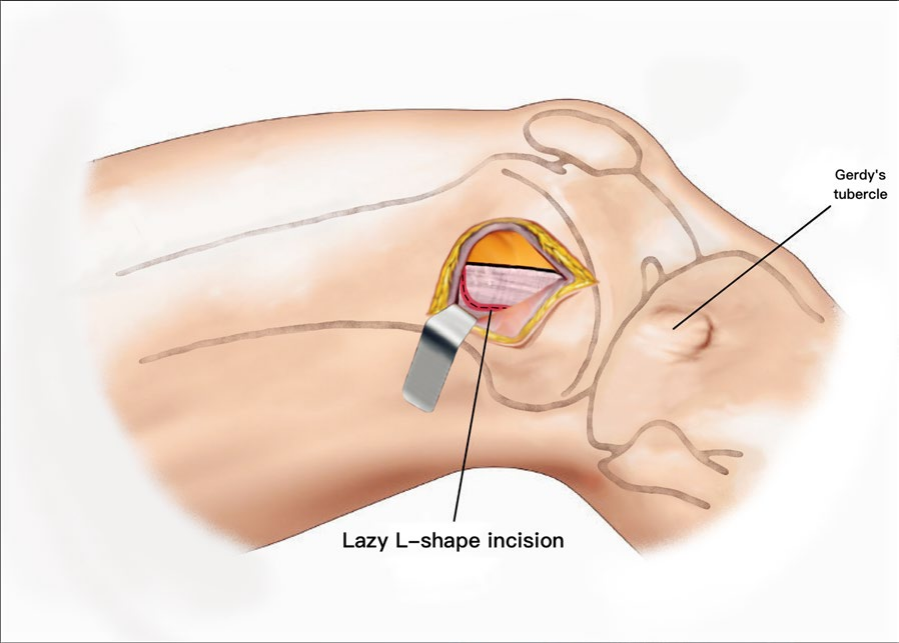
The lazy L-shape release of iliotibial band

**Fig. 6:**
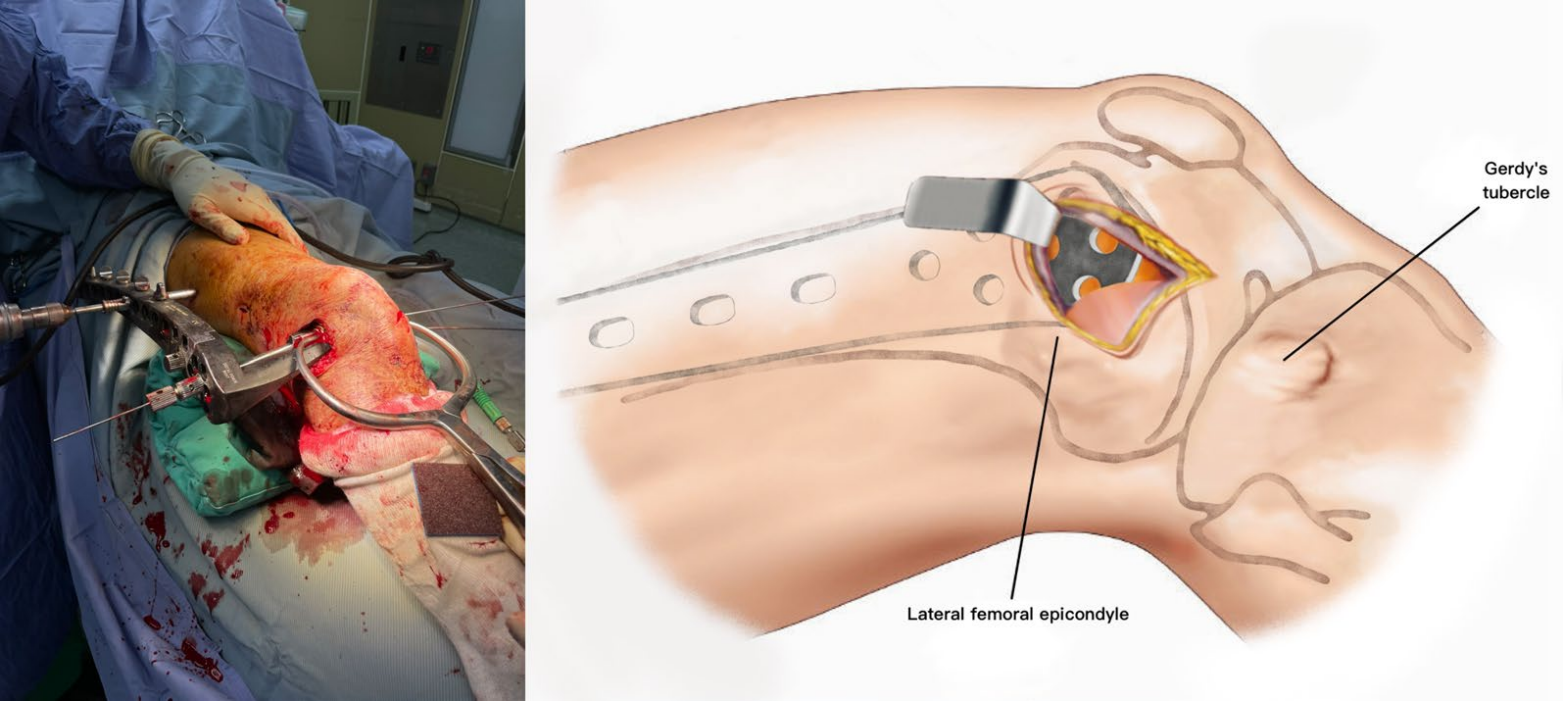
Application of the periarticular reduction clamp and K-wire to the distal fragment

**Fig. 7:**
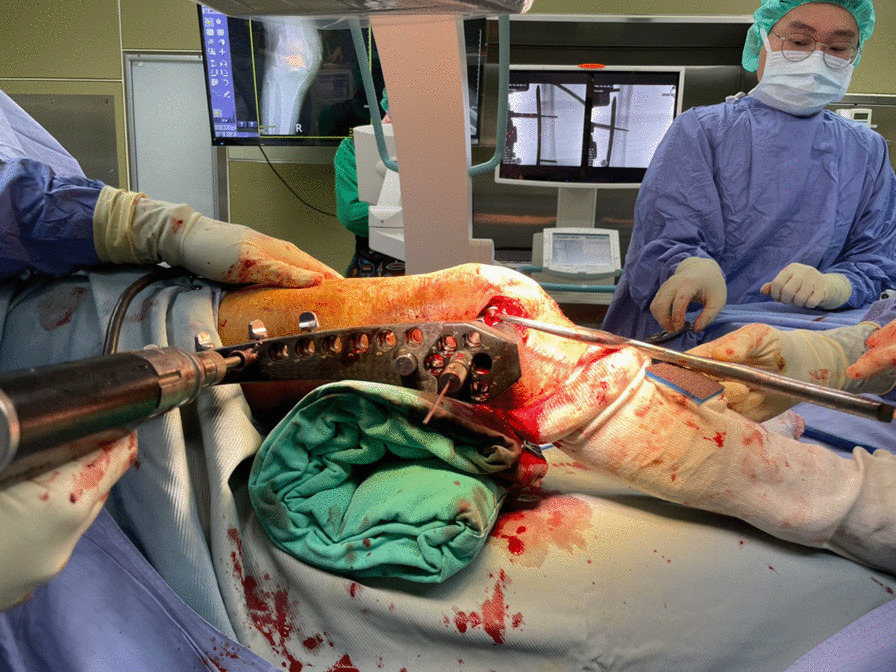
Proximal screws applied through puncture windows, each approximately 1 cm, under the guidance of intraoperative radiography

**Fig. 8:**
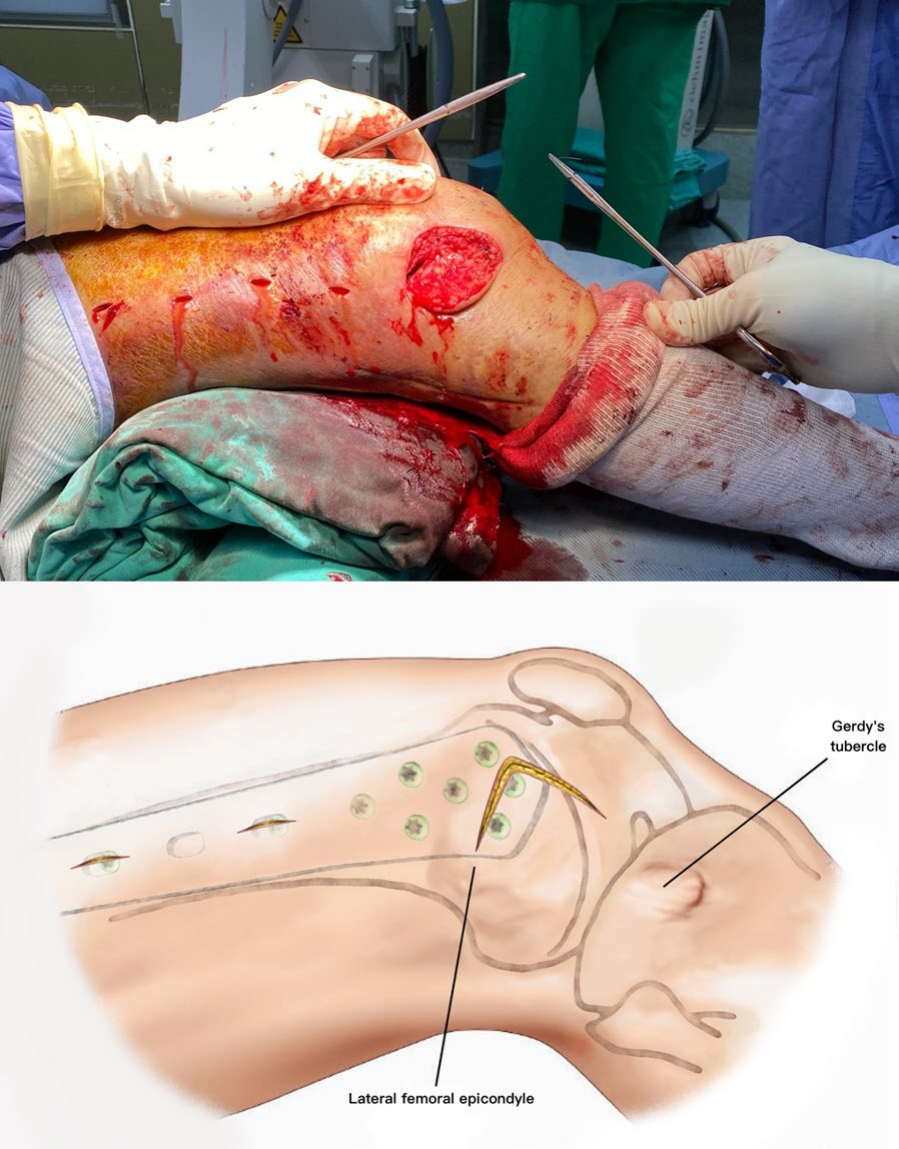
The retinaculum is closed with absorbable sutures

**Fig. 9:**
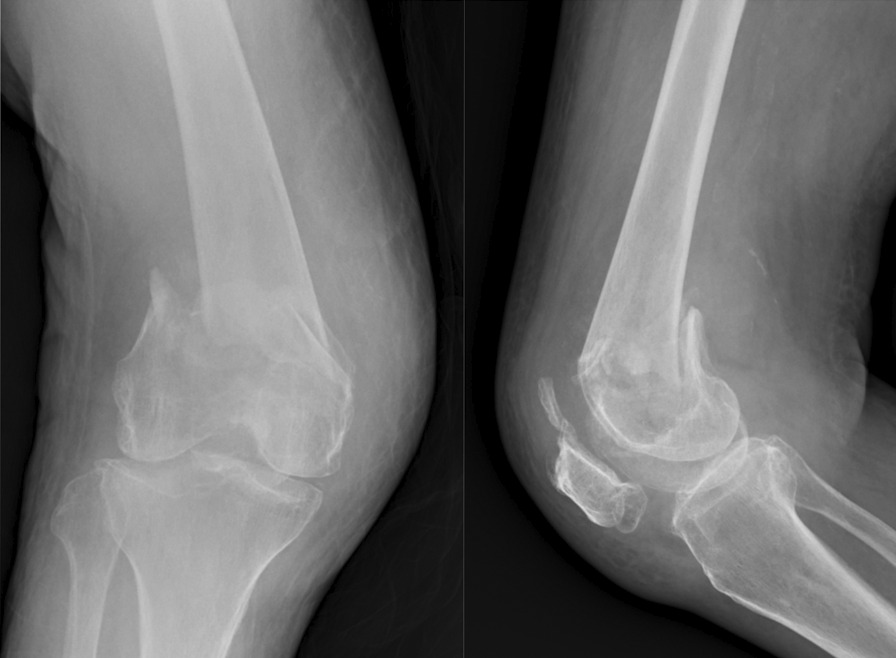
Preoperative radiograph showed distal femur intercondylar fracture

**Fig. 10:**
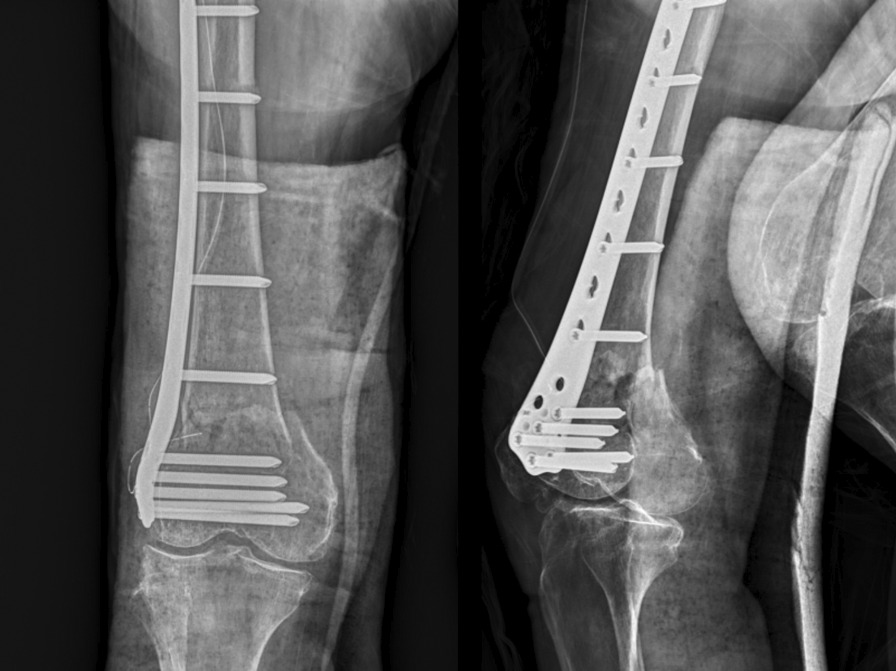
Postoperative radiograph showed well reduced alignment without joint incongruity and varus deformity

The patient is placed supine on a radiolucent table. A triangular leg roll is positioned beneath the knee to maintain a flexion of approximately 45° of both the hip and knee (Fig. 1). A tourniquet is not used because it may compress and anchor the quadriceps muscles. The incision starts from Gerdy’s tubercle and extends proximally, parallel to the patellar tendon. A perpendicular turn is made after reaching the mid-patellar level and is extended toward the lateral femoral epicondyle along the skin crease of the knee, forming a lambda-shaped incision (Fig. 2). Sharp dissection is used to develop full-thickness skin flaps. Flaps are developed only enough to visualize the underlying lateral patellar retinaculum. Adequate hemostasis is achieved at this stage through electrocauterization because a tourniquet is not used in this procedure. The retinaculum is then incised in the same manner as the skin flap, also forming a lambda-shaped arthrotomy, thereby providing access to the knee joint (Fig. 3). An Army-Navy retractor is placed to protect the extensor component as the hypertrophic suprapatellar synovium is excised entirely. The Army-Navy retractor is then replaced by a curved Hohmann retractor placed across the suprapatellar pouch to render visible the intercondylar notch and joint congruity (Fig. 4). In certain cases where the posterior femoral condyle or posterior femoral cortex needs to be further inspected, the vertical retinacular incision can be extended posteriorly as required. The upper part of iliotibial band can be released via a lazy L-shape incision if it hinders retinacular release (Fig. 5). After intercondylar fracture reduction, Kirschner wires and/or periarticular reduction clamps can be used to fix the distal fragment temporally. This is followed by the minimally invasive plate osteosynthesis procedure (Fig. 6). The periosteal elevator or rasps can be used to bluntly separate the vastus lateralis muscle from the anterolateral femoral shaft for plate insertion. The common comminuted metaphyseal segment is bypassed with the bridging technique. The proximal screws are applied through approximately 1-cm-puncture windows under the guidance of intraoperative radiography (Fig. 7). In some complex intercondylar fractures or fracture fragments at the far medial side of the femur, a small medial window of approximately 3 cm can be made above the medial epicondyle to aid in the reduction. The medial window should match the location where the periarticular reduction clamp can also be applied. After implantation, if the iliotibial band is released during the procedure, it can be repaired from distal end to proximal end by simple approximation without excessive tension. The proximal end can be let alone without repair if too much tension noted during the repair procedure. Both the retinaculum and iliotibial band are closed and repaired with an absorbable suture (we use 2–0 for the retinaculum and No. 1 for the iliotibial band; Figs. 8, 9, 10).

## Results

The demographic data are illustrated in Table 1. Twelve men and seven women (mean age: 52.5 years, range: 20–81) were included. No patient had any neurological or vascular deficits at the time of admission. All patients were treated with a single lateral plating of the distal femur. Iliotibial band release was done in seven cases. The mean operation time was 192 (90–420) min, some of which included time operating on concomitant fractures. All surgical wounds healed by first intention. No implant failure occurred in any patient. Radiographic signs of bone union were reached at a mean of 13.7 (7–22) months. Only one patient reached full union within 9 months. Most of the patients required up to at least 1 year to achieve full union. One patient sustained malunion with a genu varus deformity. No poor tendon healing in the cases of iliotibial band release was observed.

**Table 1:** Demographic data of patients undergoing the lambda-incision approach

Patient no	Sex	Age	Fracture type	Concomitant Injury	ITB release	Time of surgery (min)	Time to union	Range of motion	Sander’s functional score
1	M	38	A3		+	130	22 m	0–90	Good
2	M	47	A3		+	190	Loss	0–130	Excellent
3	M	67	C2		−	240	16 m	0–140	Excellent
4	F	54	C2		−	155	12 m	0–140	Excellent
5	F	66	C2	Distal radius fracture	−	180	11 m	0–140	Excellent
6	M	22	C2		−	180	13 m	0–110	Good
7	M	20	C2	Ipsilateral tibial plateau fracture	−	390	15 m	10–100	Good
8	M	70	C1		−	90	Loss	0–130	Excellent
9	F	61	C2	1st metacarpal fracture	+	165	12 m	0–110	Excellent
10	M	34	C3		+	165	Loss	0–130	Excellent
11	M	58	A3		−	150	12 m	0–100	Good
12	M	40	C3		+	160	7 m	10–100	Excellent
13	F	70	C2		−	150	Loss	0–100	Good
14	F	71	C2	Humeral head fracture	−	195	Loss	0–100	Good
15	F	63	C1		−	135	17 m	0–100	Fair
16	M	46	C2		+	180	Loss	10–90	Fair
17	M	33	C2	Bilateral humeral neck fracture 5th metacarpal neck fracture 2nd metatarsal neck fracture	−	420	Loss	0–130	Fair
18	M	63	C2	2nd distal phalangeal near amputation	−	300	Loss	Loss	–
19	F	59	A3		−	120	Loss	Loss	–
20	F	81	C2		+	135	Loss	0–130	Excellent
Mean		52.5 y/o				192	13.7 m	1.5–115	

Regarding the postoperative joint range of motion, eight patients had maximal flexion of > 120°, eight patients had flexion between 90° and 120°, and two patients had flexion of < 90°. Clinically, nine patients had excellent results, six had good results, three had fair or poor results, and two were lost to follow-up, leaving insufficient data for evaluation. The three unsatisfactory (fair and poor) results were due to restricted knee motion, residual pain, malunion with genu varus deformity, with one patient having another injury during the same trauma event that precluded the patient from resuming work.

## Discussion

Indirect reduction of the metadiaphysis while minimizing the damage to the peripheral soft tissue has gradually become the standard procedure in distal femur fracture repair, and this has led to predictably high rates of union without the need for grafting, with a low incidence of infection and low estimated blood loss [5–8].

The classical lateral approach described by Marcy remains popular for treating supracondylar fractures. Several modifications have been proposed to make it into a minimally invasive procedure. The arterial supply to the patella is mostly preserved when performing a lateral approach because the incision is rather distant from the genicular anastomotic safe margin [9]. However, such an approach can only provide limited access to the intercondylar notch unless the wound is extended and dissected thoroughly to subluxate the whole extensor component medially. The modified J-shaped or hockey-stick incision is less commonly used because it causes extensive soft tissue destruction.

Fractures that involve the joint are quite common. Up to 55% of distal femoral fractures have joint involvement [10]. Physicians have started to use lateral parapatellar arthrotomy, which is more familiar to orthopedic surgeons who practice TKA to gain direct access to the distal femoral joint. This incision obliterates the lateral arterial anastomosis, increasing the difficulty of planning future TKA. Krettek et al. emphasized the value of indirect reduction to avoid additional damage to the surrounding soft tissue and blood supply around the metadiaphysis [4]. Barei et al. observed that the contact of the posterior femoral cortex is strongly correlated with primary union and can even obviate the need for additional bone grafts in distal femur fractures, with some metaphysis bone loss [11]. However, it is more difficult to confirm the posterior femoral cortex reduction status through the lateral parapatellar approach.

Starr et al. argued that their swashbuckler approach can completely expose the entire distal femur while sparing the extensor mechanism and superior part of the lateral genicular anastomosis. Beltran et al. later modified this traditional swashbuckler approach to the mini-swashbuckler approach, which entails 87% joint exposure but is much more biologically friendly and avoids dissection in the area of the metadiaphysis. Chao et al. compared the postoperative results of the original and modified swashbuckler approaches and noted that the limited surgical field in the mini-swashbuckler approach may increase the operation and fluoroscopy times but significantly decreased the total blood loss [12]. Thus, the mini-swashbuckler approach has been considered an ideal technique for treating distal femur fractures.

**Fig. 11:**
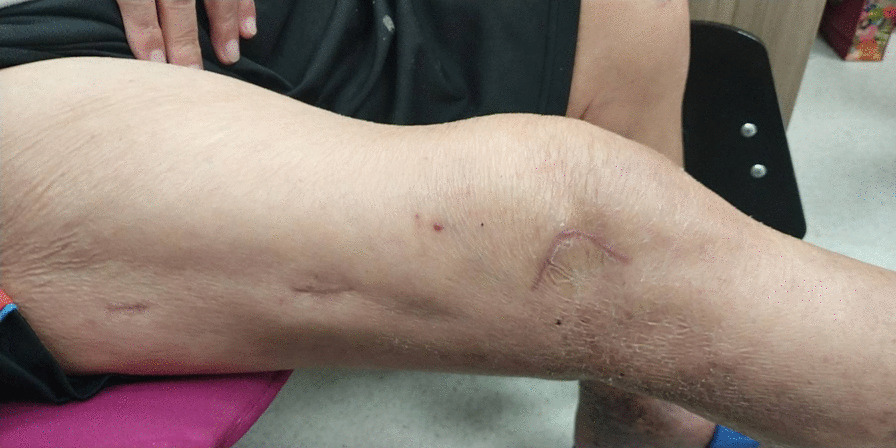
Excellent healing of the incision with a highly satisfactory cosmetic appearance

However, all the current approaches to distal femur fractures share a similar pattern: they all use a straight or lazy-curved incision adjacent to the patella or along the lateral side of the femur. A straight incision allows for easier proximal extension, but it requires a longer wound to expose the same area than that required by other shapes of incision. The shortest incision length to distal femur on record in the literature is mini-swashbuckler approach that requires only 12 cm. However, the average length of the lambda incision is around 9 cm. The current concept in treating distal femur fractures is to reduce only the articular surface, leaving the metadiaphysis portion untouched. Accordingly, we developed an approach using a lambda-shaped incision, which sacrifices the potential for proximal extension but preserves much more peripheral soft tissue. The corner flap formed by the lambda incision can be turned down, creating a much larger window than that offered by a same-length straight incision. The vertical turn at the mid-patellar level parallels the skin crease and geniculate artery, further reducing the damage to the surrounding soft tissue and resulting in a smooth healing wound and highly satisfactory cosmetic outcome (Fig. 11). Moreover, the vertical turn can expose the lateral surface of the lateral femoral condyle, making plate and distal screw insertion easier than when done using a straight incision. In certain circumstances where the posterior condyle or posterior cortex needs to be inspected, extending the vertical incision more laterally and releasing the upper part of iliotibial band via a lazy L-shape incision can easily expose this area. With the lower iliotibial band intact, the upper released iliotibial band can be repaired just by simple approximation without excessive tension. As a result, there are no poor healing of ligament and any lateral side laxity issues in our series. For a far medial fragment that is unreachable from the lambda window, we can create an additional window approximately 3 cm above the medial epicondyle and use a finger to manipulate the fragment and reduce it while viewing it through the lambda window or under fluoroscopy. The medial open wound can also be used to apply the periarticular reduction clamp to keep the joint reduced while continuing minimally invasive plate osteosynthesis.

## Limitations

The main drawback of the lambda incision is its strict vertical turn location. The vertical turn made to form the flap is what makes the joint easily accessible, but it also makes the operative field difficult to extend proximally. When proximal extension is necessary due to an inappropriate vertical turn level, it takes an especially long additional wound to make a turn in the proximal direction. An extra proximal incision of at least 5 cm may be an alternative to prevent a massive extended wound. We modified the ideal incision site under fluoroscopy, and through experience, we arrived at the current incision landmark. Second, the surgeon must be used to operating through a mobile window to reduce fractures. Both Dr. Wu and Dr. Lin have been performing minimally invasive surgery at different fracture sites for years and are already used to this. For beginners, we strongly recommend starting with the traditional lateral parapatellar incision and gradually adopting techniques to minimize the wound, finally arriving at the lambda incision. Last, this approach can prolong the operation time compared with other approaches because of the extremely limited visual field. When we started performing the lambda incision, the operation took 2–4 h depending on fracture complexity. However, over years of practice, the operation time has been minimized to within 2 h in most cases. Accordingly, the patient’s hemodynamic status should be thoroughly evaluated and monitored when deciding whether to use this technique.

## Conclusions

Minimally invasive locking plate osteosynthesis is the current preferred treatment for patients with distal femur fractures. In our experience, the minimally invasive lambda-incision approach allows sufficient visual access to almost the entire joint surface, including the entire lateral femoral condyle, trochlear surface, and distal medial condyle, where reduction is required when performing minimally invasive plate osteosynthesis. The metadiaphysis region is mostly left untouched, thus maintaining the best fracture-healing environment (Table 2). In the future, we aim to compare the outcome of the lambda-incision approach with other types of approaches to distal femur fractures.

**Table 2:** Advantages and disadvantages of the lambda-incision approach

Advantages	Disadvantages
The least metadiaphyseal soft tissue damage Preserve all the anastomosis ring Well exposure of the implant position site Posterior femoral cortex reachable The best cosmetic appearance	Long learning curve More surgical time Difficult to extend the wound if clinically required

## Data Availability

The datasets used and/or analyzed during the current study are available from the corresponding author on reasonable request.

## Abbreviation

TKA: Total knee arthroplasty

## References

[1] Marcy GH (1947). The posterolateral approach to the femur. J Bone Jt Surg Am.

[2] Olerud S (1972). Operative treatment of supracondylar–condylar fractures of the femur. Technique and results in fifteen cases. J Bone Jt Surg Am.

[3] Starr AJ, Jones AL, Reinert CM (1999). The ‘‘Swashbuckler’’: a modified anterior approach for fractures of the distal femur. J Orthop Trauma.

[4] Krettek C, Muller M, Miclau T (2001). Evolution of minimally invasive plate osteosynthesis (MIPO) in the femur. Injury.

[5] Ostrum RF, Geel C (1995). Indirect reduction and internal fixation of supracondylar femur fractures without bone graft. J Orthop Trauma.

[6] Kregor PJ, Stannard JA, Zlowodzki M, Cole PA (2004). Treatment of distal femur fractures using the less invasive stabilization system. Surgical experience and early clinical results in 103 fractures. J Orthop Trauma.

[7] Nayak RM (2011). Minimally invasive plate osteosynthesis using a locking compression plate for distal femoral fractures. J Orthop Surg (Hong Kong).

[8] Weight M, Collinge C (2004). Early results of the less invasive stabilization system for mechanically unstable fractures of the distal femur (AO/OTA types A2, A3, C2, and C3). J Orthop Trauma.

[9] Lazaro LE, Cross MB, Lorich DG (2014). Vascular anatomy of the patella: implications for total knee arthroplasty surgical approaches. Knee.

[10] Court-Brown CM, Caesar B (2006). Epidemiology of adult fractures: a review. Injury.

[11] Barei DP, Beingessner DM (2012). Open distal femur fractures treated with lateral locked implants: union, secondary bone grafting, and predictive parameters. Orthopedics.

[12] Xiang C, Jiang K, Chen Q, Li Y, Bai H, Chen L (2019). Early effectiveness of mini-Swashbuckler approach for distal femoral type C fractures. Chin J Reparative Reconstr Surg.

